# Acute total body ionizing gamma radiation induces long-term adverse effects and immediate changes in cardiac protein oxidative carbonylation in the rat

**DOI:** 10.1371/journal.pone.0233967

**Published:** 2020-06-04

**Authors:** Elliot Rosen, Dmitry Kryndushkin, Baikuntha Aryal, Yanira Gonzalez, Leena Chehab, Jennifer Dickey, V. Ashutosh Rao

**Affiliations:** Center for Drug Evaluation and Research, Office of Biotechnology Products, United States Food and Drug Administration, Silver Spring, Maryland, United States of America; Helmholtz Zentrum München, GERMANY

## Abstract

Radiation-induced heart disease presents a significant challenge in the event of an accidental radiation exposure as well as to cancer patients who receive acute doses of irradiation as part of radiation therapy. We utilized the spontaneously hypertensive Wistar-Kyoto rat model, previously shown to demonstrate drug-induced cardiomyopathy, to evaluate the acute and long-term effects of sub-lethal total body gamma irradiation at two, four, and fifty-two weeks. We further examined irreversible oxidative protein carbonylation in the heart immediately following irradiation in the normotensive Wistar-Kyoto rat. Both males and females sustained weight loss and anemic conditions compared to untreated controls over a one-year period as reflected by reduced body weight and low red blood cell count. Increased inflammation was detected by elevated IL-6 serum levels selectively in males at four weeks. Serum cardiac troponin T and I analyses revealed signs of cardiomyopathy at earlier timepoints, but high variability was observed, especially at one year. Echocardiography at two weeks following 5.0Gy treatment revealed a significant decrease in cardiac output in females and a significant decrease in both diastolic and systolic volumes in males. Following 10.0Gy irradiation in the normotensive Wistar-Kyoto rat, the heart tissue showed an increase in total protein oxidative carbonylation accompanied by DNA damage indicated by an increase in γ-H2AX. Using proteomic analyses, we identified several novel proteins which showed a marked difference in carbonylation including those of mitochondrial origin and most notably, cardiac troponin T, one of the key proteins involved in cardiomyocyte contractility. Overall, we present findings of acute oxidative protein damage, DNA damage, cardiac troponin T carbonylation, and long-term cardiomyopathy in the irradiated animals.

## Introduction

Total body irradiation (TBI) can lead to the acute radiation syndrome (ARS) which affects the hematopoietic, gastrointestinal, and neurovascular systems in a dose dependent fashion. ARS consists of four phases spanning hours to weeks including prodromal, latent, manifest illness, and final. The prodromal phase symptoms can be mild or intense depending on exposure level and include fever, skin irritation, nausea, vomiting, and headache. This period is followed by a latent phase, lasting hours to weeks, where symptoms subside but this is accompanied by a reduction in lymphocytes and granulocytes. The latent phase is followed by symptoms of illness related to the hematopoietic, gastrointestinal, and neurovascular syndromes depending on the exposure level. In the final phase, the intensity of the exposure, total absorbed dose, and heterogeneity will determine if the patient survives [1]. Subjects exposed to sub-lethal levels of TBI are subsequently at risk for long-term complications involving growth, endocrine, cardiopulmonary, cardiovascular, renal, ocular, and central nervous system effects [2, 3].

Reports on survivors of Hiroshima-Nagasaki, Chernobyl, and Fukushima as well as patients that received radiation therapy for cancer have shown increased cardiovascular disease with significantly higher cardiac mortality than expected [4–9]. Additional research intended to provide insight into the pathophysiology of cardiac radiation injury could allow for more informed therapeutic interventions to be utilized.

While radiation-induced heart damage has been well-documented in the clinical setting [10] and in rat models [11–13], protein oxidation by carbonylation of specific cardiac proteins has not been explored thoroughly in this context as a mechanism for cardiomyopathy. Though the mechanism of Radiation-Induced Heart Disease (RIHD) is known to include excess formation of reactive oxygen species and long-term oxidative modifications, protein oxidation endpoints have not been thoroughly explored in this context [14–16]. Recent reports by Azimzadeh et al. have considered overall heart protein carbonylation in a mouse model [17] as well as a population of nuclear facility workers diagnosed with radiation-induced ischemic heart disease. The authors describe a general increase in carbonyl content in pooled heart tissue samples of subjects diagnosed with the disease and estimated to be exposed to greater than 0.5Gy [18].

Protein carbonylation is an irreversible post-translational modification, marking proteins for proteasomal degradation. Moreover, protein carbonylation can result in unfolding or changes to the protein structure and thereby function [19]. We hypothesized that the oxidative damage resulting from sub-lethal TBI could be monitored in the myocardium by protein carbonylation and correlated with a release of cardiac troponins.

We selected the spontaneously hypertensive strain of Wistar-Kyoto rat (SHR) to evaluate the acute and chronic effects of gamma TBI as it closely models the drug-induced cardiomyopathy associated with anthracyclines, a class of antineoplastic drugs indicated for a variety of cancers [20]. Our group has previously reported anthracycline-induced carbonylation of cardiac myosin binding protein C in the SHR model [21]. Others have previously used the SHR model for studying RIHD and other radiation-induced injuries [22, 23]. We chose to evaluate this well-characterized model for its utility as an irradiation-induced model of cardiomyopathy and related protein oxidation. We also confirmed the mechanism of protein oxidation by carbonylation in the heart utilizing the normotensive Wistar-Kyoto rat to ensure that the mechanism is consistently present in the less-sensitive heart model.

## Materials and methods

### Animals

To evaluate the acute and chronic effects of total body gamma irradiation, we used twelve-week-old Wistar-Kyoto-derived spontaneously hypertensive rats (SHR), obtained from Envigo (Indianapolis, IN). Cohorts of ten animals per group were monitored for chronic effects underwent necropsy after two, four, or fifty-two weeks. For monitoring acute biochemical changes, we used the parent Wistar-Kyoto strain, obtained from the same source. This cohort of five animals per group underwent necropsy after twenty-four hours. Animals were separated by gender and housed two animals per cage for social well-being and provided with appropriate items for environmental enrichment in an environmentally controlled room (18–21°C, 40–70% relative humidity, 12h light/dark cycle). Animals were fed Certified Purina Rodent Chow #5002 (Ralston Purina Co., St. Louis, MO) and water ad libitum. The Institutional Animal Care and Use Committee, Center for Biologics Evaluation and Research, U.S. Food and Drug Administration prospectively approved the experimental protocol (ASP WO-2011-11). The protocol was performed in an AAALAC-accredited facility by AALAS-certified laboratory animal technicians. All procedures for animal care and housing complied with the Guide for the Care and Use of Laboratory Animals of the National Institutes of Health. Animals were monitored twice daily for experimental endpoint criteria and euthanized immediately if the following was observed: tumor size greater than 4cm^3^, body weight loss greater than 20%, appearance of skin lesions, inability to reach food or water, difficulty breathing, or signs of pain/distress. Nine animals from the one-year cohort met the criteria for euthanasia before fifty-two weeks. One animal, from the 0.5Gy dose, one-year cohort, died at week fifty-one with unknown cause of death before the end of the planned necropsy at week fifty-two. Euthanasia and animal sacrifice were carried out by exsanguination via the inferior vena cava under isoflurane anesthesia. Blood serum was separated from the blood samples collected at necropsy before storage at -80°C. The heart was weighted, sectioned horizontally into upper part (atria) and lower part (ventricles), flash frozen, and stored at -80°C.

### Irradiation

Total body gamma irradiation of spontaneously hypertensive rats given sub-lethal (0.5, 3.0, and 5.0Gy) and normotensive Wistar-Kyoto rats given lethal irradiation (10.0Gy) was produced with a Gammacell 40 Cs-137 irradiator (MDS Nordion, Mississauga, ON). The dose rate range of the irradiator was determined to be 64–68 cGy/min using Gafchromic EBT-XD dosimetry film (Bridgewater, NJ) that is placed in the middle of two high density polyethylene panels, performed annually. Four unanesthetized animals were placed in a plastic irradiation container with individual compartments at one time. Animals in the control group were sham-irradiated for the same periods of time as needed for irradiations.

### Serum cTnI, cTnT, and IL-6 quantification

Cardiac troponin I in the bloodserum was measured using a quantitative immunoassay involving paramagnetic microparticle solid phase capture technique on the Erenna instrument (MilliporeSigma, Burlington, MA) as per manufacturer’s instructions. Cardiac troponin T measurement was performed by Boston Children’s Hospital, CERLab, using the Troponin T hs STAT, a quantitative sandwich enzyme immunoassay on the Roche E Modular system (Roche Diagnostics, Indianapolis, IN). An ELISA kit specifically for rat was used to measure IL-6 (Thermo Fisher Scientific, Waltham, MA).

### Echocardiography

Echocardiogram measurements were performed with the Vevo2100 instrument, and data analysis was performed with the accompanying Vevo LAB software, v1.7.0 (FUJIFILM VisualSonics, Toronto, Canada). M-mode measurements were recorded with the MS250 transducer (20 MHz) in the parasternal short-axis view in triplicate. All measurements were performed on lightly-anesthetized animals (1.5–2.0% isoflurane) while maintaining a heart rate of 350–400 beats per minute. The body temperature of the animal was maintained with a heated stage.

### Protein carbonylation and γ-H2AX by western blot

Left ventricle heart tissue was sectioned into small pieces and gently homogenized with a tissue grinder on wet ice in radioimmunoprecipitation assay (RIPA) buffer (MilliporeSigma, Burlington, MA) containing protease (Roche Diagnostics Corporation, Inc., Indianapolis, IN) and phosphatase inhibitors (MilliporeSigma, Burlington, MA). Tissue homogenates were allowed to rest for 20 minutes before centrifugation at 12,000 g for 20 minutes at 4°C. Supernatant was transferred to new Eppendorf tube before protein quantification by BCA assay (Thermo Scientific, Rockford, IL) and storage at -80°C. We derivatized protein carbonyls with DNPH using a protocol based on earlier publications [21, 24, 25]. A total volume of 15μL protein lysate, including 10μg of protein and 6% (w/v) SDS, was prepared for each sample. We then added 15μL of 20mM DNPH in 10% (v/v) TFA before incubating at room temperature for ten minutes. Neutralization of the reaction was achieved by adding 15μL of 2M Tris in 30% (v/v) glycerol containing 7% (v/v) β-mercaptoethanol. Lastly, two identical 4–12% bis-tris gels (Thermo Fisher Scientific, Waltham, MA) were loaded in duplicate with 3.3 μg of DNP-derivatized sample per lane. The proteins from the first gel were transferred to an immobilon-P PVDF membrane (MilliporeSigma, Burlington, MA) and probed for specific protein carbonylation while the second gel was used for Coomassie staining. The membrane was blocked for one hour (LI-COR Biosciences, Lincoln, NE) and incubated with goat polyclonal anti-DNP primary antibody (Cat. A150-117A, 1:1,000 dilution, Bethyl Laboratories, Montgomery, TX) followed by donkey anti-goat IRDye 800CW secondary antibody (LI-COR Biosciences, Lincoln, NE). Western blot analysis for γ-H2AX was performed using mouse monoclonal anti-γ-H2AX (Cat. 05–636, 1:500 dilution, MilliporeSigma, Burlington, MA), while uniform protein loading was monitored using a rabbit polyclonal anti-GAPDH antibody (Cat. NB100-56875, 1:500 dilution, Novus, Centennial, CO). An Odyssey infrared imaging system (LI-COR Biosciences, Lincoln, NE) was used to analyze western blots and Coomassie stained gels. For carbonyl quantification, the entire lane for protein carbonyl signal in the western blot was normalized with the corresponding Coomassie stained lane in the gel.

### Protein identification

Equal amounts of non-irradiated and irradiated cardiac tissue lysate were treated with 10mM DNPH for 10 minutes at room temperature. Proteins were precipitated with 20% trichloroacetic acid (TCA) for 15 minutes on ice. Precipitated proteins were separated by centrifugation, washed with ethanol/ethyl acetate mixture (1:1 v/v) and dissolved in rehydration buffer composed of 7M urea, 2M thiourea, 2% CHAPS, 0.5% ampholytes pH 3–10. Protein concentration was determined by Bradford protein assay. For isoelectric focusing, 30 μg of DNP-derivatized protein was applied per 7 cm IPG strip. After isoelectric focusing, IPG strips were used to run second dimensional gel electrophoresis using 4–12% gel. Gel staining and western blot were performed using the procedure described earlier with samples being run in duplicate, one for Coomassie gel staining and one to be transferred to a membrane for protein carbonylation. The proteins in gel were transferred to an immobilon-P PVDF membrane and processed for western blotting using anti-DNP antibody. Coomassie-stained protein spots corresponding with proteins that showed a change in carbonylation level on the membrane were excised from the gel.

The samples were digested with sequencing grade modified trypsin (Promega Corporation, Madison, WI) for 16 hours at 37°C. Liquid chromatography-tandem mass spectrometry analysis was performed on trypsin-digested peptides using an Ultimate LC and Fusion Orbitrap MS (Thermo Fisher, San Jose, CA). Samples were then loaded onto a PepMap, C18, 5μm, 0.3x5 mm trap cartridge (Thermo Fisher, San Jose, CA). Next, samples were eluted onto a reversed phase PepMap, C18, 3μm, 100A, 15cm x 75μm I.D. Easy-Spray column (Thermo Fisher, San Jose, CA) with a linear 60-min gradient of acetonitrile (2–62%) containing 0.1% formic acid at a flow rate of 250μL/min. Finally, the peptides were sprayed into the Fusion Orbitrap. The data dependent acquisition mode was enabled and each FTMS MS1 scan (60,000 resolution) was followed by LTQ MS2 scans allowed to maximize sampling rates. The ion transfer tube temperature was 250°C. The spray voltage was 1.8kV. Trypsin digested bovine serum albumin was used as a quality control sample for the analysis. Peptide search was performed with Proteome Discoverer 1.4 (Thermo Fisher Scientific, Waltham, MA) in combination with the Swiss-Prot rat SP database to match MS/MS spectra to peptides using trypsin specificity. A precursor tolerance of 25ppm and fragment ion tolerance 0.6Da were employed. Carbamidomethylation at cysteine and variable modifications consisting of oxidation of methionine and deamidation of glutamine/asparagine were fixed modifications considered. Significance of spectra/peptide matches were allowed with at least 5% false discovery rate and proteins must have had at least two unique peptides.

For quantifying the fold change in carbonylation, three biological replicates for both non-irradiated and 10Gy-irradiated animals were run simultaneously (total of six membranes). An Odyssey infrared imaging system (LI-COR Biosciences, Lincoln, NE) was used to capture images and spot intensity. The average fold change in carbonyl level in irradiated samples was calculated relative to the non-irradiated samples.

### Two-color, 2D-gel electrophoresis

We used 2D-gel electrophoresis followed by a two-color western blot analysis to confirm the selective carbonylation of cardiac troponin T (cTnT). Heart tissue lysates were treated with DNPH using a previously published protocol [26]. Protein derivatization and western blot were performed using the procedure mentioned earlier for protein identification. The PVDF membrane was incubated with two primary antibodies for three hours at room temperature, a goat polyclonal anti-DNP antibody (Cat. A150-117A, 1:1,000 dilution, Bethyl Laboratories, Montgomery, TX) and a mouse monoclonal antibody against cTnT (Cat. sc-33721, 1:200 dilution, Santa Cruz Biotechnology, Dallas, TX). After three PBST washes, the membrane was incubated for one hour at room temperature with donkey anti-goat 800CW (green) secondary antibody for carbonyl and donkey anti-mouse 680LT (red) secondary antibody for cTnT. Finally, the membrane was scanned with the Odyssey infrared imaging system (LI-COR Biosciences, Lincoln, NE) and the extent of carbonylation of cTnT was confirmed with the overlapping green and red bands on the membrane. Experiment was performed on four biological replicates and the level of carbonylation of cTnT was normalized to total cTnT protein present and expressed as a fold change in carbonyl content of irradiated to non-irradiated samples.

### Statistical analyses

Statistical analyses were performed using the software Prism 6 v6.07 (GraphPad Software, La Jolla, CA). A two-way ANOVA with Dunnett’s test to correct for multiple comparisons was performed for body weight, troponin, and CBC analyses. A Student’s *t*-test was performed to evaluate the change in carbonylation of troponin of the western blot membrane. A p value of ≤ 0.05 was considered statistically significant for all analyses. Fourteen proteins identified as exhibiting a change in carbonylation reflect an average of three biological replicates.

## Results

### Total body acute and chronic effects of sub-lethal gamma irradiation

**Fig 1:**
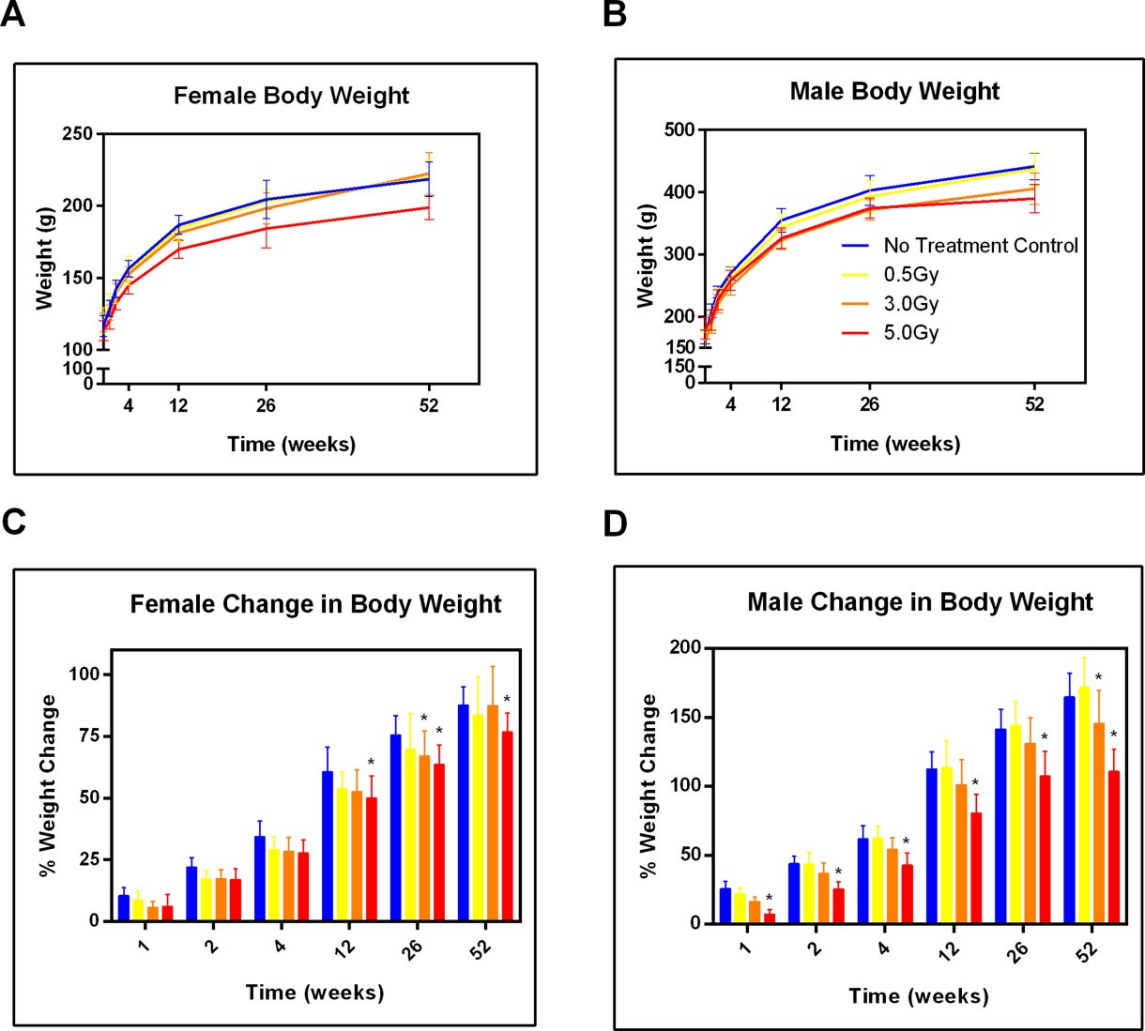
Total body irradiation negatively impacts growth for up to one year. The average female (A) and male (B) body weight is displayed over one year. The average change in female (C) and male (D) body weight is displayed over one year. From left to right, groups of bars denote no treatment control, 0.5Gy, 3.0Gy, and 5.0Gy for each timepoint. Error bars represent standard deviation. NT = no treatment; *p<0.05 wrt NT, two-way ANOVA with Dunnett’s test to correct for multiple comparisons; n = 10.

The general health of the spontaneously hypertensive rats was monitored over a one-year period by measuring body weight (Fig 1), clinical signs of toxicity, and hematological indices to inform both acute and chronic effects of sub-lethal total body irradiation (TBI). Starting one-week post irradiation, males receiving 3.0Gy TBI showed significantly reduced body weight with respect to control cohort for each timepoint up to one year. Males receiving 5.0Gy showed a significant weight change with respect to control starting at week one. In terms of chronic effects, both male and female cohorts receiving 5.0Gy TBI showed inhibited weight gain. Females receiving 5.0Gy showed a significant weight change with respect to control starting at week twelve while males receiving either 3.0Gy or 5.0Gy showed a significantly less body weight than control from week 12 up to one year.

The animal cohort followed for one year was closely monitored for multiple clinical observations including development of a scruffy coat, lethargic and/or hunched appearance, abdominal fluid, weight loss, tumor development, and early death (Table 1). Most notably, two male animals receiving 5.0Gy TBI were euthanized early at 32 and 51 weeks due to signs of sickness including scruffy coat, lethargic/hunched appearance, and >20% weight loss in accordance with our animal study protocol. At necropsy, this same group had multiple animals presenting with abdominal fluid and increased incidence of tumors. The tumors were mostly subcutaneous, located on the back or under arms/legs. One was found intertwined with intestine at necropsy. One of the subcutaneous tumors and the tumor found in the gut were sent to a pathologist for further evaluation. The pathologist determined the subcutaneous tumor to be a carcinoma that likely arose from the mammary gland but was not able to exclude adnexal structures and skin as possible sites of origin. The tumor found in the gut was described as small intestine and lymph node that were effaced by a malignant neoplasm with rhabdoid-like neoplastic cells whose site of origin is unknown.

**Table 1:** General clinical observations one year after irradiation.

Females	Clinical Signs of Toxicity					
Radiation Dose	Scruffy Coat	Lethargic / Hunched Appearance	Abdominal Fluid	Weight Loss (>20%)	Tumor Development	Early Death or Euthanasia
No Treatment			1			
0.5Gy			1			
3.0Gy					3	3
5.0Gy					3	1
Males	Clinical Signs of Toxicity					
Radiation Dose	Scruffy Coat	Lethargic / Hunched Appearance	Abdominal Fluid	Weight Loss (>20%)	Tumor Development	Early Death or Euthanasia
No Treatment						
0.5Gy			4			1
3.0Gy	1	1	1	1	3	3
5.0Gy	2	2	4	3	7	2

n = 10 animals per group

The complete blood count measured at 52 weeks after TBI reflects a sustained deficiency in white blood cell count in males receiving 5.0Gy TBI (Table 2). Sex-specific differences in white blood cell count in the untreated groups are consistent with what has been reported for the Sprague-Dawley strain [27]. Significantly lower red blood cell counts were observed in the 3.0Gy and 5.0Gy TBI male cohorts. Both male and female cohorts receiving the 5.0Gy TBI, and the male cohort receiving 3.0Gy TBI, showed significantly lower red blood cell counts after one year. Multiple other metrics including hemoglobin level, mean corpuscular volume, mean corpuscular hemoglobin concentration, and red cell distribution width in the male, 3.0Gy TBI group showed statistically significant change with respect to control, while the respective female group showed signs of recovery by the one-year timepoint.

**Table 2:** Total body irradiation results in sustained complete blood count abnormalities, 52 weeks after TBI.

** **			White Blood Cell	Red Blood Cell	Hemoglobin	Hematocrit	Mean Corpuscular Volume	Mean Corpuscular Hemoglobin	Mean Corpuscular Hemoglobin Conc.	Red Cell Distribution Width	Platelet Count
			WBC	RBC	HGB	HCT	MCV	MCH	MCHC	RDW	PLT
		Units	10^3^/uL	10^6^/uL	g/dL	%	fL	pg	g/dL	%	10^3^/uL
**Male**	**NT**	**AVG**	8.0	9.28	14.2	37.0	39.8	15.2	38.3	23.5	637
		**SD**	1.2	0.51	0.8	1.8	0.5	0.4	0.7	0.9	33
	**0.5Gy**	**AVG**	7.1	9.01	14.0	36.5	40.6	15.6	38.4	23.6	675
		**SD**	1.1	0.27	0.7	1.2	1.7	1.1	0.9	1.0	61
	**3.0Gy**	**AVG**	6.3	8.32*	13.2*	35.4	42.8*	15.9	37.2*	21.5*	691
		**SD**	2.2	0.75	0.6	1.4	2.8	0.8	0.5	1.3	58
	**5.0Gy**	**AVG**	5.6*	7.65*	12.2*	33.2*	43.4*	16.0*	36.9*	20.3*	692
		**SD**	1.4	0.52	0.6	1.8	0.9	0.4	0.4	1.1	134
**Female**	**NT**	**AVG**	5.0	8.2	13.7	36.2	43.9	16.6	37.8	19.9	644
		**SD**	1.0	0.4	0.6	1.2	0.9	0.4	0.6	0.6	60
	**0.5Gy**	**AVG**	4.7	8.0	13.2	35.3	44.8	16.7	37.4	19.9	579
		**SD**	1.3	1.2	1.6	3.8	3.5	1.0	0.8	0.8	102.7
	**3.0Gy**	**AVG**	5.5	8.18	13.5	36.5	44.7	16.5	37.0*	19.7	525*
		**SD**	1.2	0.50	0.7	1.5	1.0	0.3	0.5	1.0	50
	**5.0Gy**	**AVG**	5.4	7.62*	12.6*	34.4*	45.1*	16.5	36.6*	18.9*	579
		**SD**	1.8	0.35	0.6	1.3	0.9	0.2	0.5	0.5	103

NT = no treatment

*p<0.05; n = 10 animals per group

Hematology analysis was performed at necropsy for animal cohorts at all three-time points. The female cohorts at two weeks showed significant decreases in red blood cell count, hemoglobin, and hematocrit at all levels of TBI, and these decreased levels were sustained at four weeks in the 3.0Gy and 5.0Gy groups (S1 Table). A significantly lower white blood cell count was observed in the 3.0Gy and 5.0Gy dose groups at two weeks that resolved by four weeks. Interestingly, males at two weeks did not show significant decreases in red blood cell count, hemoglobin, and hematocrit at 0.5Gy and 3.0Gy TBI. By four weeks however, males receiving 3.0Gy TBI showed decreased red blood cell count and hemoglobin. The data suggest that females have an earlier onset radiation-induced anemia (negative erythrogenic response) compared to males.

### Acute inflammatory response in rats following irradiation

**Fig 2:**
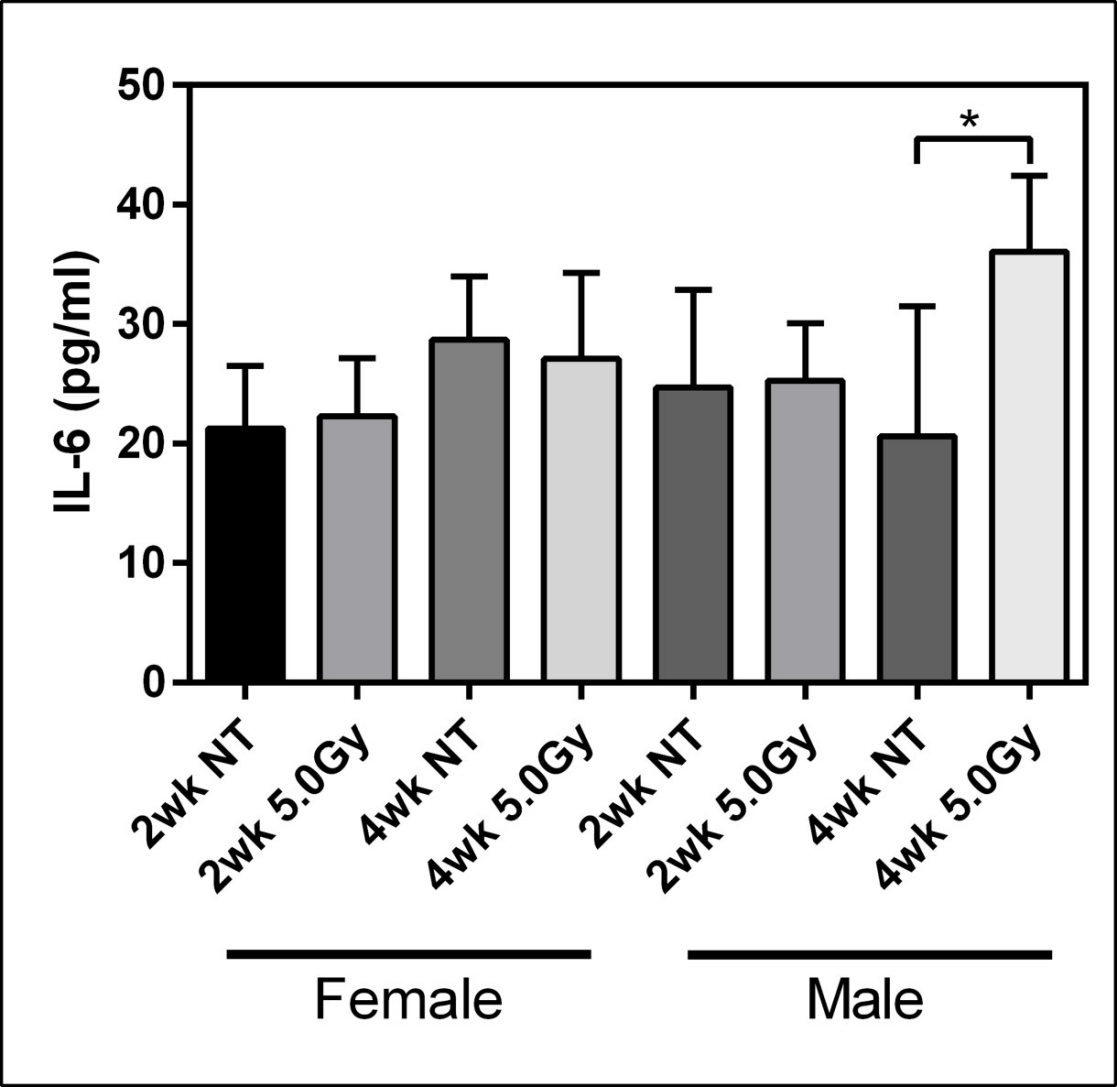
Inflammatory response following total body irradiation. Serum levels of IL-6 at two and four weeks after 5.0Gy total body irradiation. Error bars represent standard deviation. NT = no treatment; n = 3.

Circulating levels of cytokine IL-6 were measured in blood sera to observe the acute pro-inflammatory response to TBI (Fig 2). Male and female cohorts receiving the 5.0Gy TBI were compared to respective control groups at both two and four weeks. A statistically significant increase in IL-6 was confirmed in males, but not females, at four weeks post exposure.

### Acute and chronic cardiomyopathy in irradiated male and female rats

**Fig 3:**
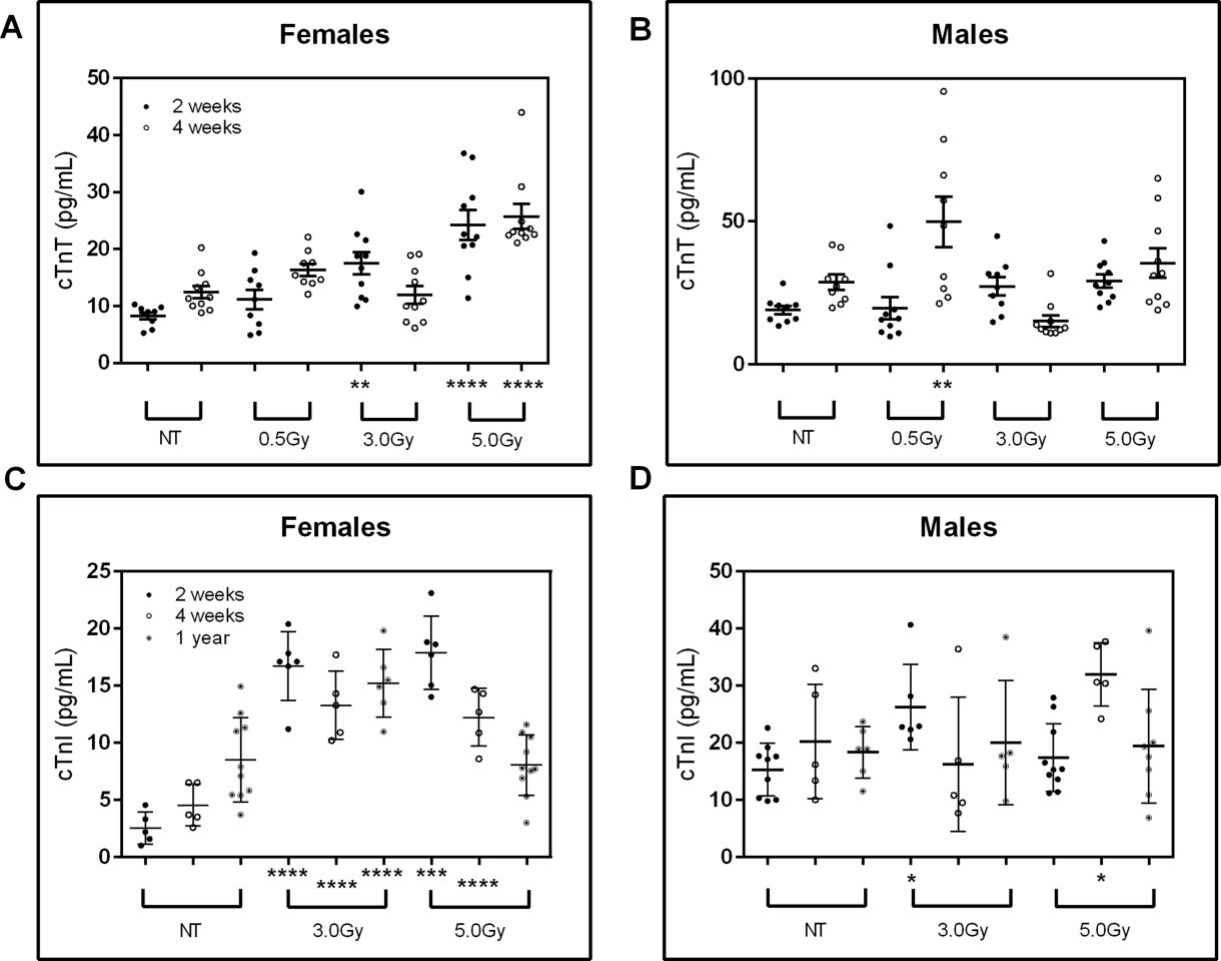
Serum levels of cTnT and cTnI reveal acute cardiomyopathy and long-term abnormalities. Increases in serum cTnT were detected in (A) female and (B) male at two- and four-weeks following irradiation. Serum cTnI levels at necropsy for (A) female and (B) male animals at two, four, and fifty-two weeks following total body irradiation. Bars represent standard error of the mean. NT = no treatment; *p<0.05, **p<0.01, ***p<0.001, and ****p<0.0001 wrt NT; n = 5–10.

Serum levels of cardiac troponin T (cTnT) were measured at two and four weeks in spontaneously hypertensive rats receiving 0.5Gy, 3.0Gy, and 5.0Gy TBI to evaluate acute cardiomyopathy (Fig 3A and 3B). Serum levels of subunit cardiac troponin I (cTnI) were also measured at the two and four-week marks, as well as one year to evaluate chronic cardiomyopathy (Fig 3C and 3D). The cTnT levels in females were significantly increased at two weeks in both 3.0Gy and 5.0Gy TBI exposure groups as well as at four weeks for 5.0Gy TBI revealing the acute effect of TBI on the heart. The male cohorts showed more variability with multiple animals showing elevated levels of cTnT and a statistically significant increase at four weeks with 0.5Gy TBI.

Cardiac troponin I levels in females were significantly increased in the 3.0Gy and 5.0Gy TBI groups at two weeks and four weeks, mirroring cTnT levels. At one year, the 3.0Gy TBI group continued to reflect statistically significant, elevated cTnI levels in females. The 5.0Gy TBI group did not reflect an elevation with respect to the control group in females. While several individual male animals exhibited elevated levels of cTnI in both near and long term, only the cohorts of 3.0Gy TBI at two weeks and 5.0Gy TBI at four weeks showed a statistically significant increase due to greater variability observed in male cohorts.

**Fig 4:**
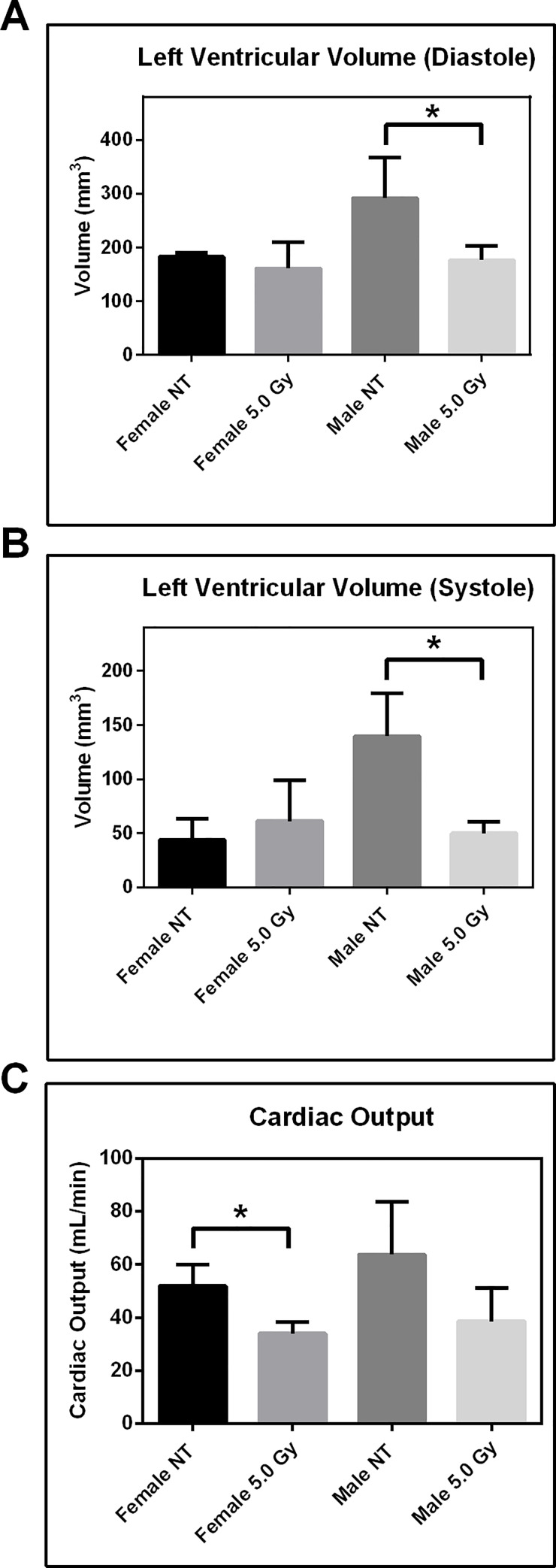
Total body irradiation results in physiological changes to heart. Echocardiography at two weeks documenting left ventricular volume at (A) diastole and (B) systole. (C) Cardiac output in males and females. Bars represent standard deviation. NT = no treatment; n = 3; *p<0.05 one-tailed Student’s *t*-test.

Further evidence of acute cardiomyopathy was collected by echocardiography, two weeks post-irradiation (Fig 4). Radiation-induced changes in cardiac function in rodent models have been reported months after radiation exposure [28]. While two weeks post-irradiation is an early timepoint to expect changes in cardiac function, we hypothesized that the oxidative molecular events that lead to cardiac function changes would be observed at these timepoints. It is worth noting that the hypertensive rat model was employed for this longitudinal component of the study precisely because this animal strain is recognized as more sensitive to doxorubicin-induced cardiac damage, and closely reflecting drug-induced cardiomyopathy in humans. Males receiving the 5.0Gy TBI showed a statistically significant reduction in left ventricular volume both at diastole and systole. Both females and males showed a reduction in cardiac output with 5.0Gy TBI, females being statistically significant. It should be noted that the male rat heart is larger in size than the female heart.

### Oxidative protein carbonylation and DNA damage in myocardium samples from 10Gy irradiated rats

**Fig 5:**
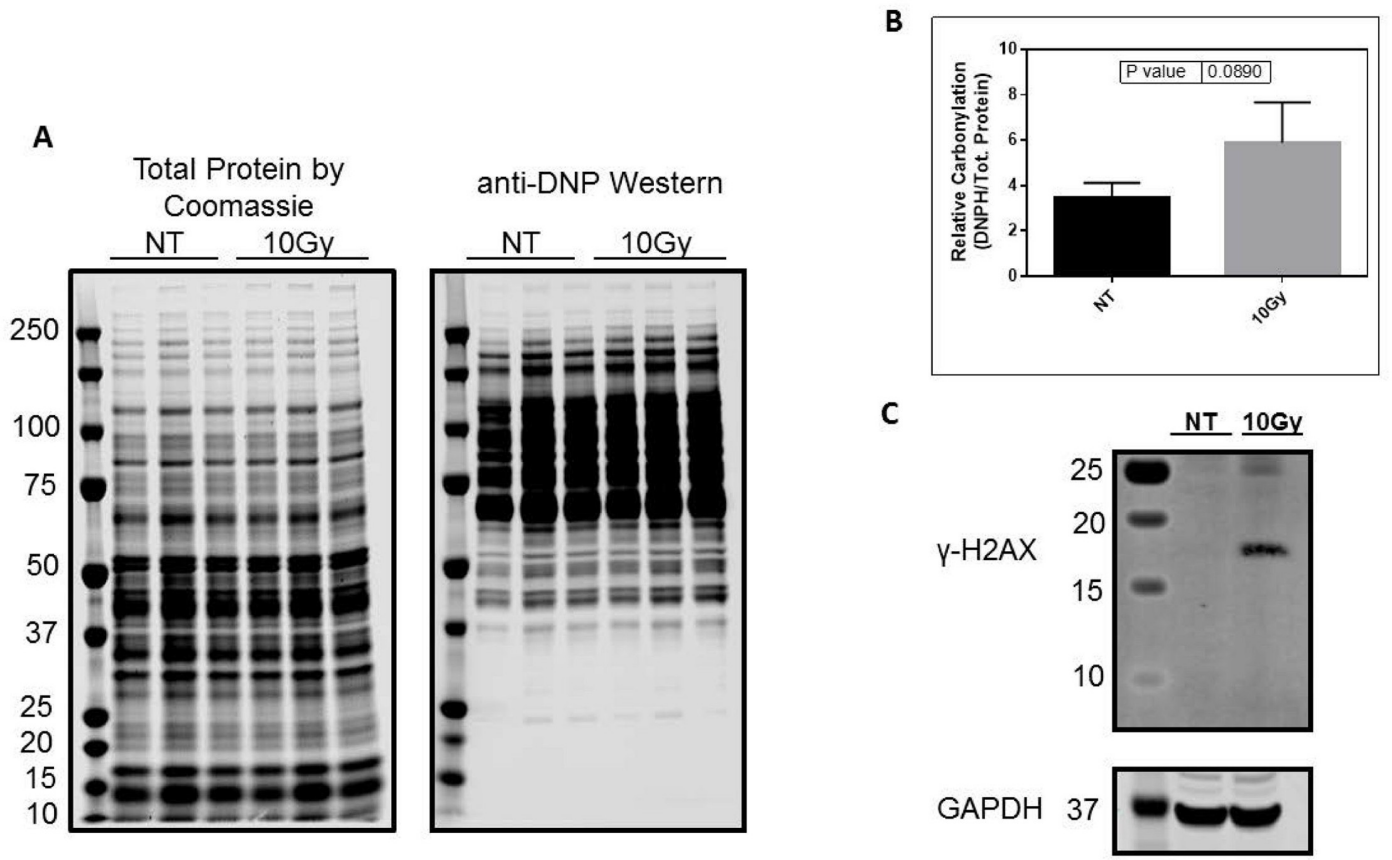
Carbonylation of proteins and DNA damage in the heart. (A) Western blot and (B) densiometric quantification for anti-DNP showing carbonylation profile of heart proteins 24hrs after 10.0Gy total body irradiation (n = 3). (C) Western blot analysis for γ-H2AX in heart tissue two hours after 10.0Gy total body irradiation. NT = no treatment.

To evaluate the acute effects of TBI on heart protein oxidative modification by carbonylation, we probed heart tissue lysates of normotensive Wistar-Kyoto rats by western blot for 2,4-dinitrophenylhydrazone (DNP)-labeled carbonyls. Twenty-four hours following 10.0Gy TBI, a significant increase in overall heart tissue protein carbonylation was observed (Fig 5A and quantified in Fig 5B). This significant increase in protein carbonyl modification was driven by increased carbonylation of proteins in the 50–250 kDa range and is accompanied by a dramatic increase in the DNA double-strand break marker γ-H2AX (Fig 5C).

### Proteomic analysis of cardiac tissue for carbonylated proteins

**Fig 6:**
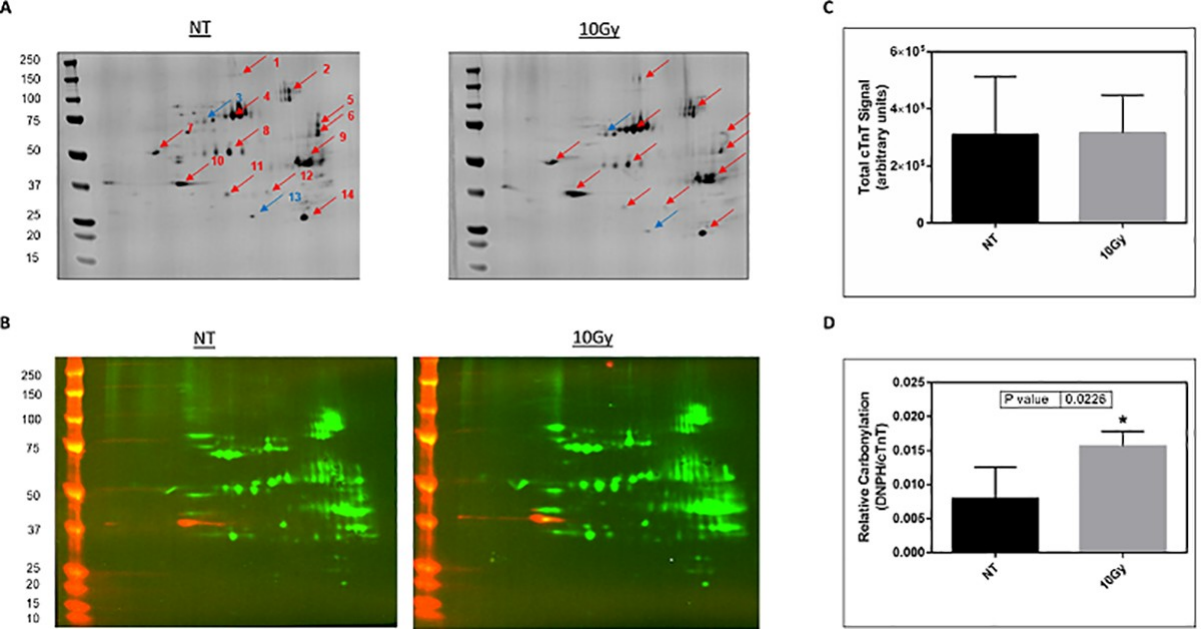
Carbonylation of proteins in male rat heart twenty-four hours after total body irradiation. (A) Two-dimensional fluorescence western blotting of protein carbonylation in no treatment (left) and 10.0Gy total body irradiation (right). Red arrows denote an increase in carbonylation in response to total body irradiation while blue arrows signify a decrease in carbonylation level. See Table 3 for protein identities and fold changes in carbonylation level. (B) Two-color, two-dimensional fluorescence western blotting showing overlap of cTnT (red) and DNP (green) signals. (C) Densitometry quantification of total cTnT protein in the rat heart. (D) Densitometry quantification of relative carbonylation of cTnT protein in the rat heart. Carbonylation signal (DNP) is normalized by cTnT. NT = no treatment; n = 4.

To further assess the nature and specificity of protein carbonylation in the heart, we performed 2D-PAGE analysis, once again probing for DNP to identify fourteen unique proteins displaying a change in protein carbonylation level (Fig 6A). These fourteen proteins were subjected to liquid chromatography–tandem mass spectrometry for identification (Table 3). Among fourteen proteins, cardiac troponin T (cTnT) was of particular interest and selected for further analysis. Elevated cardiac troponin T detected in the blood is a well-documented biomarker for cardiac damage [29]. The identity of troponin T was further confirmed by overlapping bands (green and red) in two-color western blotting using specific antibodies against DNP (green) and troponin T (red), (Fig 6B). Twenty-four hours following 10.0Gy TBI, there was no change in total cTnT protein level (Fig 6C), yet there was a statistically significant increase in carbonylation of cTnT in rat myocardium compared to non-treated control (Fig 6D). Since saline perfusion may not fully remove blood from the heart tissue, it is expected to see proteins commonly found in the blood such as serum albumin and serotransferrin.

**Table 3:** Selected proteins from 2D-PAGE gel of male rat myocardium exhibiting change in carbonylation pattern following irradiation.

Spot ID	Carbonyl Fold Change	Identity	Known Function
1	3.6	Serum albumin	Maintain oncotic pressure, carrier protein
2	1.9	Serotransferrin	Iron binding, transport
3	-0.9	60 kDa heat shock protein	Maintain imported protein folding
4	1.5	Serum albumin	Maintain oncotic pressure, carrier protein
5	1.9	ATP synthase subunit alpha	ATP synthesis and ion transport
6	1.7	ATP synthase subunit alpha	ATP synthesis and ion transport
7	1.8	ATP synthase subunit beta	ATP synthesis and ion transport
8	2.0	Alpha-enolase	Enzyme: glycolysis, glycolysis, growth control, hypoxia tolerance and allergic response
9	1.2	Serum albumin	Maintain oncotic pressure, carrier protein
10	3.6	Troponin T, cardiac	Cardiac structural protein
11	1.6	Isocitrate dehydrogenase [NAD] subunit alpha	Catalyzes the decarboxylation of isocitrate
12	1.7	Electron transfer flavoprotein subunit alpha	Accepts electrons from several mitochondrial dehydrogenases
13	-0.6	Heat shock protein beta-6	Cardiac myocyte contractility
14	3.1	Heat shock protein beta-5	Molecular chaperone, HSP

Fourteen spots were selected for MS analysis, and 11 unique proteins were identified as showing a change in carbonylation level after twenty-four hours following 10.0Gy TBI. The findings represent the average of three biological replicates.

## Discussion

This report highlights the acute and chronic effects of TBI in the rat with a focus on changes in cardiac-specific protein oxidation due to irreversible carbonylation. Elevated levels of cardiac troponins detected in the sera signaled acute cardiomyopathy suggesting that a mechanism involving oxidative protein carbonylation of cardiac troponin T (cTnT) may coincide with proteasomal degradation and/or subsequent release of this protein into the blood. Indeed, elevated levels of cTnT is currently used as a biomarker of cardiac injury and its release into the blood is associated with radiotherapy in breast cancer patients [30]. The long-term chronic effects of TBI can be observed by the reduced growth of animals and sustained deficiencies of hematology markers. To the best of our knowledge, the carbonylation of cardiac troponin T following irradiation or in response to any cardiotoxic agent has not been previously reported.

Previous reports have described carbonylation of cellular proteins in bone marrow [31], cardiac tissue [17, 32], and specifically cardiac-mitochondrial [33] proteins in mice in response to TBI. While these previous studies have not looked at individual heart protein carbonylation status, they agree with our finding that total, overall carbonylation is increased and changes to protein levels of mitochondrial origin occur rapidly in response to acute TBI. In the rat, changes in protein carbonylation in multiple tissues including skeletal muscle [34] and liver [35] have been reported in response to TBI. Interestingly, increases in protein carbonylation have also been detected in non-irradiated cells in close proximity to irradiated cells (bystander cells) and progeny of irradiated cells in response to ionizing radiation as a result of increased oxidative stress [14]. It should also be mentioned that extensive protein acetylation/deacetylation of heart proteins has been found in response to TBI [36, 37].

The use of the spontaneously hypertensive and normotensive Wistar-Kyoto rat strains as models for monitoring chronic and acute irradiation-induced cardiomyopathy respectively, is unique to this study. We want to highlight that the two rat models are different, so conclusions can be carefully drawn, taking this into account. We elected to use the SHR model for the initial longitudinal study as it has been demonstrated as a good model for studying the kinetics and molecular events associated with free-radical mediated and doxorubicin-induced cardiomyopathy. The purpose of the initial long-term study was to track the overall physiological changes (body weight, CBCs, echocardiography, etc.). The acute timepoints were included to investigate the potential early biochemical changes that occur immediately post irradiation that lead to the chronic conditions observed in the first study. By employing the normotensive rat with a higher irradiation exposure, we exclude the sensitivity of the hypertensive rat and focus on generating a robust oxidative stress response with the 10Gy exposure. The release of troponins into the bloodstream is currently considered a sensitive biomarker of myocardial damage. Our ability to simultaneously analyze troponins in the bloodstream and protein carbonylation in the myocardium of the same animal allows us to understand the mechanistic correlation between cardiomyopathy and protein oxidation by carbonylation. Our novel finding that increased levels of carbonylation of cTnT occur within 24hrs in response to TBI and this event coincides with elevated cTnT levels in serum two and four weeks later, leads us to hypothesize that protein carbonylation may play a role in the mechanism of irradiation-induced cardiomyopathy and related cardiac muscle contraction dysfunction. We hypothesize that carbonylation of cTnT results in its displacement from the contractile machinery of the cardiomyocyte and subsequent release from its complex with other proteins responsible for muscle contraction. It is also likely that this oxidation event signals for proteasomal degradation of damaged proteins, and under more stressful conditions, oxidized proteins leak out of the cardiomyocyte into the bloodstream where they can be readily detected even at low levels [38, 39]. While it has been proposed that the initial release of troponin into the blood is from the cytosolic pool, followed by a sustained release over days due to the gradual breakdown of the myofibrils [40], some evidence suggests that some fraction of cTnT may be bound in equilibrium based on the cTnT:tropomyosin binding constant and readily released following myocardial insult [41]. Continuing with downstream degradation and excretion, reports by Streng et al. have described the subsequent degradation of cTnT by thrombin in the bloodstream [42] and appearance in urine following acute myocardial infraction [43]. We have previously shown that oxidative carbonylation of cardiac myosin binding protein C (cMyBP-C) results in loss of association with actin, proteasomal degradation, and actin-binding related functional impairment. Also of note, in the context of chronic disease, new evidence suggests that cardiac contractile proteins including cMyBP-C and troponins may behave as antigens contributing to autoimmunity in the context of cardiovascular disease [44]. Further studies are needed to determine the exact nature of the dissociation event and downstream effects of troponin T carbonylation as we observed in our group’s experience with cMyBP-C [21].

The most susceptible residues for carbonylation are lysine (K), arginine (R), threonine (T), and proline (P) [45]. It has been reported that tryptophan residue 161 of the troponin I subunit in fast-twitch skeletal muscle in a rat model for acute oxidative stress can become carbonylated in response to x-ray irradiation [46]. Interestingly, Magherini et al. found the rat skeletal troponin T isoform to be naturally carbonylated and reduced in response to acute swimming exercise, which was theorized by the authors to be due to further oxidation to carboxylic acid, decarbonylation via a thiol-dependent reduction, or protein degradation via ubiquitination [47]. Collectively, these pieces of evidence may be indicative of selective carbonylation and ensuing degradation of the oxidized protein. It should be noted that the cardiac isoforms of troponin I and T are distinctly different from skeletal muscle, yet highly conserved across species. No site-specific carbonylation of amino acid residues on cardiac troponin T have been reported, but multiple amino acid residues susceptible to carbonyl modification are present in troponin T (S1 Fig). Lysine, arginine, threonine, and proline residues are more susceptible to carbonylation, and when these residues cluster together, the likelihood increases further [48].

While this is the first report of cTnT carbonylation, other post translational modifications, such as phosphorylation, methionine oxidation, and glycosylation have been previously reported for cardiac troponin T, I, and C subunits present in myocardial contractile machinery [49–51]. The irreversible oxidative carbonylation can act as an alternative mechanism by which the protein may be dissociated from its complex in the myocardium and subsequently released into the blood. Additional studies are needed to determine the site-specificity, kinetics, reversibility, and clinical impact of cardiac troponin T carbonylation. Collectively, this study establishes cardiac troponin T oxidation by carbonylation as a potential mechanistic link between TBI-induced cardiomyopathy and release of troponins into the bloodstream.

## Supporting information

S1 Table Complete blood count abnormalities observed at two and four weeks after irradiation. NT = no treatment; *p<0.05; n = 10 animals per group.
(TIF)

S1 Fig Amino acid sequence of rat cTnT. The amino acid sequence of adult rat cTnT is featured. Specific amino acid residues susceptible to carbonylation are underlined.
(TIF)

S1 Raw Images (PDF)

## Abbreviations

TBI: Total body irradiation
ARS: Acute radiation syndrome
RHID: Radiation-Induced Heart Disease
SHR: spontaneously hypertensive rat
cTnT: cardiac troponin T
cTnI: cardiac troponin I
DNP: 2,4-dinitrophenylhydrazone
cMyBP-C: cardiac myosin binding protein C
