# Supplementary figures and images for: Acute total body ionizing gamma radiation induces long-term adverse effects and immediate changes in cardiac protein oxidative carbonylation in the rat
Source: PLoS One. 2020 Jun 4;15(6):e0233967. doi: 10.1371/journal.pone.0233967 (PMC7272027; doi:10.1371/journal.pone.0233967)

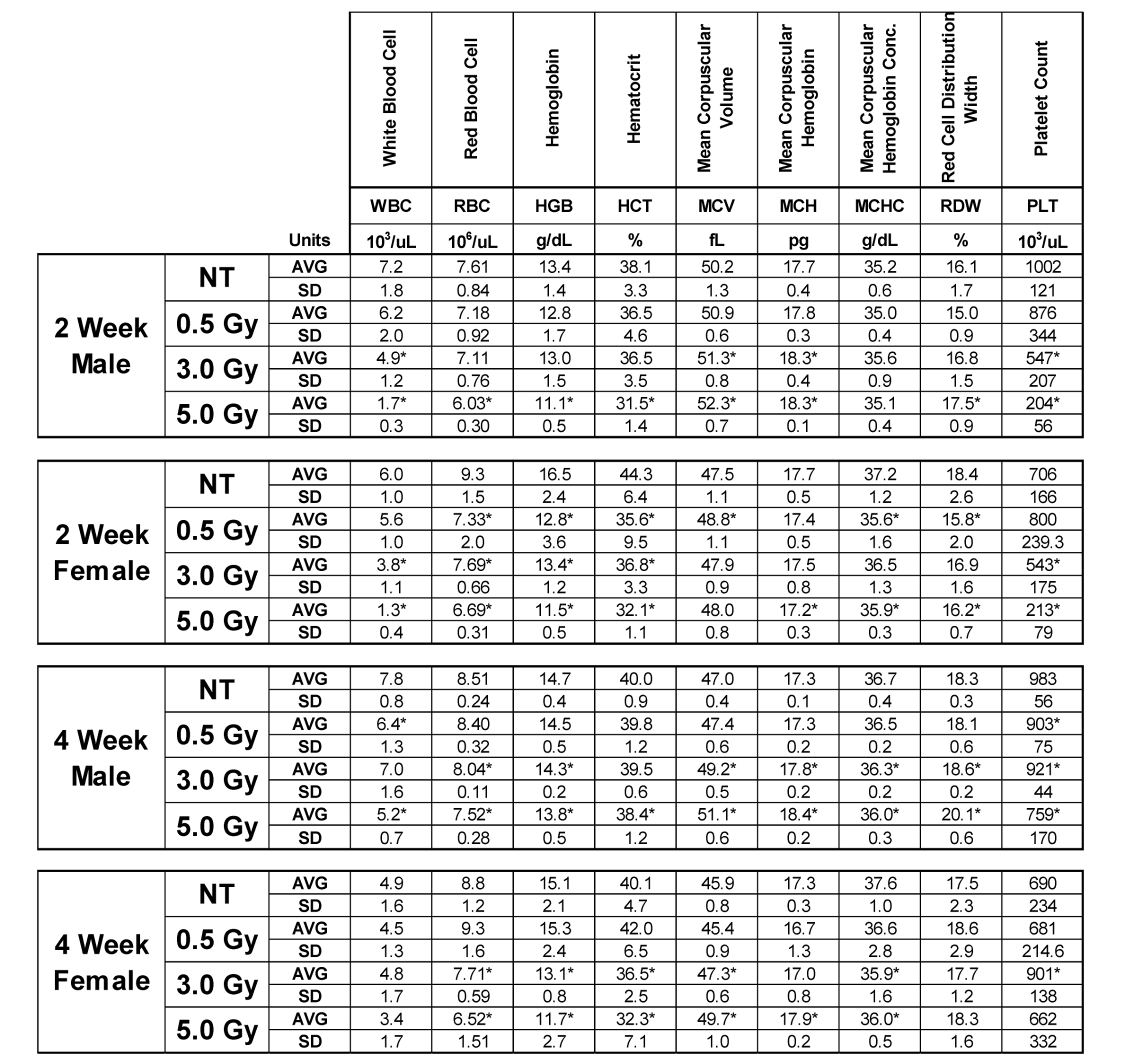

Supplement: S1 Table — NT = no treatment; *p<0.05; n = 10 animals per group. (TIF) [file pone.0233967.s001.tif]

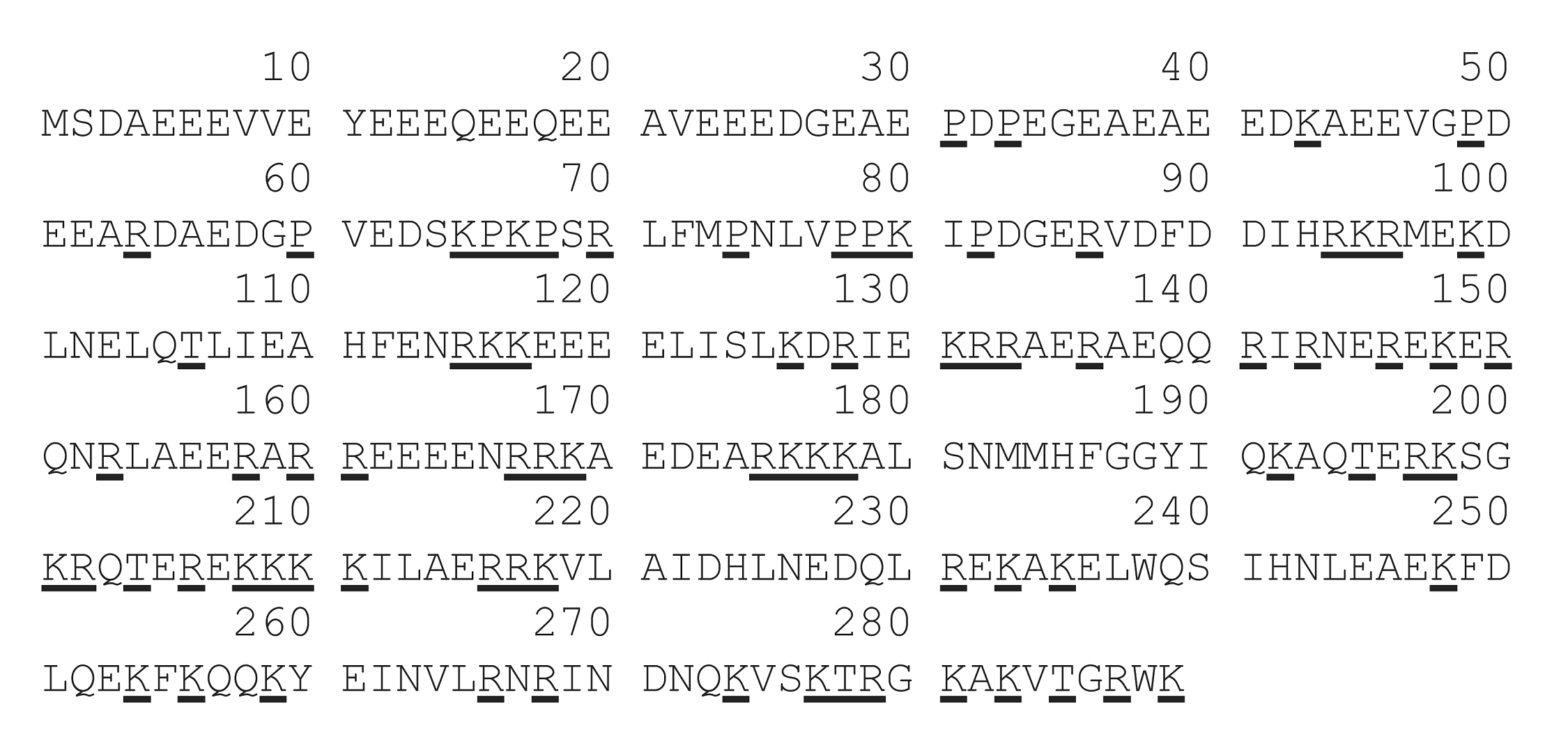

Supplement: S1 Fig — The amino acid sequence of adult rat cTnT is featured. Specific amino acid residues susceptible to carbonylation are underlined. (TIF) [file pone.0233967.s002.tif]
