# Supplementary material for: Acute total body ionizing gamma radiation induces long-term adverse effects and immediate changes in cardiac protein oxidative carbonylation in the rat
Source: PLoS One. 2020 Jun 4;15(6):e0233967. doi: 10.1371/journal.pone.0233967 (PMC7272027; doi:10.1371/journal.pone.0233967)

Two-Dimensional, Two-Color Western Blot for DNPH (Green) and cTnT (Red)

Sample number indicated in white

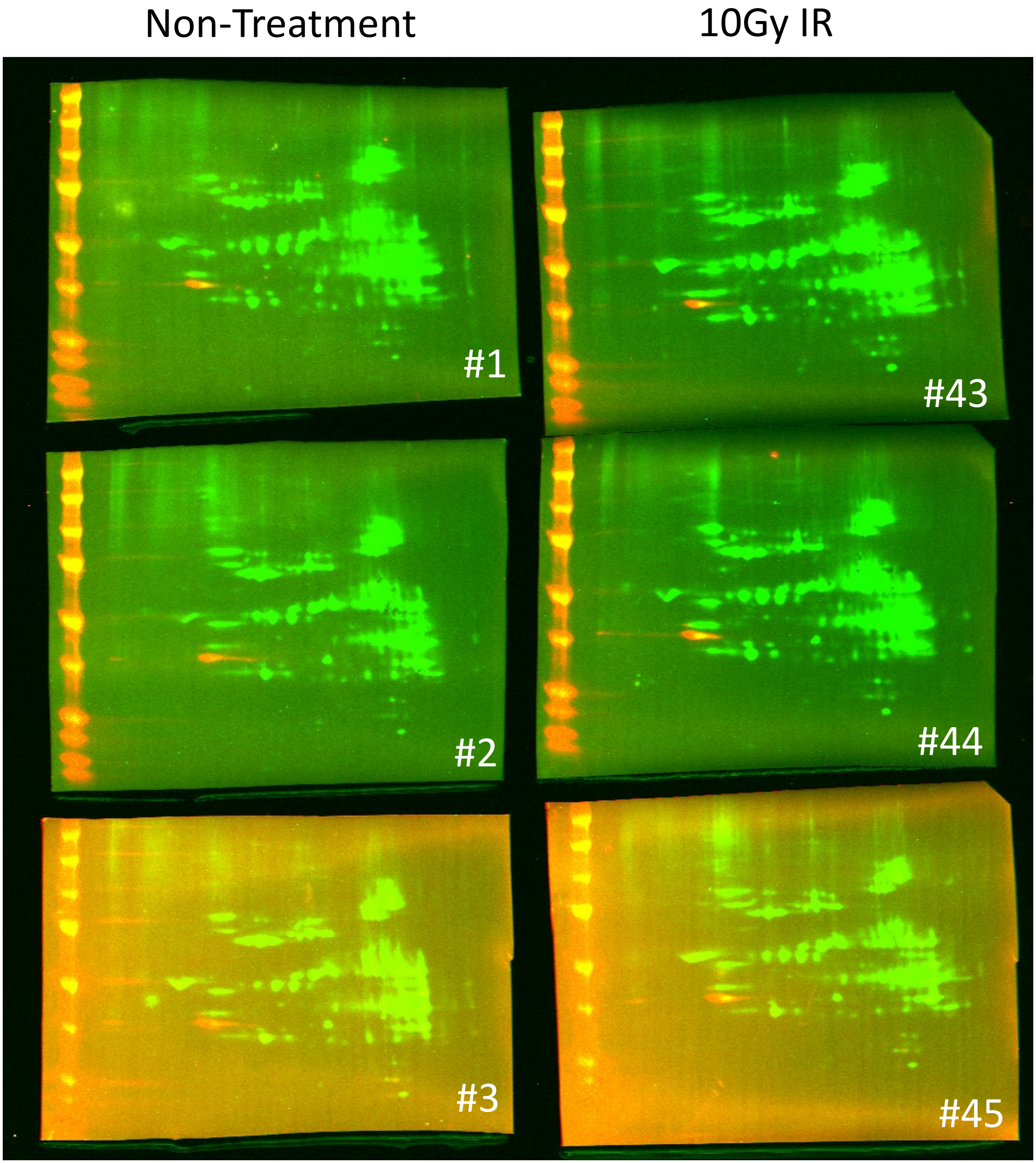

Supplement: S1 Raw Images — (PDF) [file pone.0233967.s003.pdf]
